# Psychotropic drug use among older people with major neurocognitive disorder: a cross-sectional study based on Swedish national registries

**DOI:** 10.1007/s00228-021-03241-7

**Published:** 2021-11-04

**Authors:** Jonas Kindstedt, Maria Sjölander, Hugo Lövheim, Maria Gustafsson

**Affiliations:** 1 Department of Integrative Medical Biology, Umeå University, SE-901 87 Umeå, Sweden; 2 Department of Community Medicine and Rehabilitation, Umeå University, SE-901 87 Umeå, Sweden

**Keywords:** Older people, Psychotropic drugs, Neurocognitive disorders, Drug utilization

## Abstract

**Aim:**

Psychotropic medications include many drugs that may be inappropriate for older individuals with cognitive impairment. In Sweden, many people become registered in the Swedish Dementia Registry when they are diagnosed with major neurocognitive disorder (NCD). In this study, we aim to describe psychotropic drug use and associated factors among older Swedish people with major NCD.

**Methods:**

This study included 38,251 people ≥ 65 years from the Swedish registry for cognitive/dementia disorders diagnosed during 2007–2017. Drug use was defined as one or more filled prescription(s) recorded in the Swedish Prescribed Drug Register during 1 July to 31 December 2017. Associations between psychotropics and age, sex, diagnosis date, Mini-Mental State Examination score and major NCD subtype were analysed through multiple logistic regression.

**Results:**

We found that 12.0% of the individuals filled at least one prescription for antipsychotics, 22.0% for anxiolytics, 23.0% for sedatives or hypnotics, 43.2% for antidepressants and 56.7% for antidementia drugs. In brief, psychotropic use was associated with female sex, higher age, longer time since diagnosis and specific subtypes of major NCD; the strongest association was found between antipsychotics and Lewy body dementia (odds ratio 2.40, 95% confidence interval 2.04–2.82).

**Conclusion:**

Psychotropic drugs were frequently dispensed among older Swedish people with major NCD. The use of antipsychotics and medications with sedative properties warrants concern, especially among those with Lewy body dementia who are severely sensitive to antipsychotics. A more restrictive prescribing pattern regarding these medications might reduce the risk of drug-related problems in this vulnerable group of people.

**Supplementary Information:**

The online version contains supplementary material available at 10.1007/s00228-021-03241-7.

## Introduction

Medications among older people often include psychotropic drugs, and a substantial proportion of these medications negatively alter dopamine or acetylcholine signalling and thus becomes especially problematic among individuals with major neurocognitive disorder (NCD) [1, 2], formerly termed dementia. Moreover, there are several burdensome neuropsychiatric symptoms (NPS) that commonly emerge over time and vary significantly between major NCD subtypes [3–5]. Consequently, the treatment recommendations for NPS differ depending on the combination of symptoms present but often involve psychotropic drugs [6, 7]. A high prevalence of psychotropics has previously been documented among older people with major NCD living in Swedish specialized residential care units and nursing homes, institutions where both first- and second-generation antipsychotics have routinely been utilized in the treatment of NPS [8, 9]. The risk of extrapyramidal symptoms and other adverse effects associated with antipsychotics are now well-documented, and these drugs should therefore be considered with extreme caution in people with major NCD [10–14]. Importantly, even short-term exposure to atypical antipsychotics is linked to a considerably higher risk of mortality and unplanned hospital admissions among these individuals [15–17]. Regarding other psychotropics, benzodiazepines are associated with both falls and delirium among older people [18, 19]. Similarly, sedating antihistamines (e.g. propiomazine and hydroxyzine) increase the risk of daytime sedation and anticholinergic side effects [20]. Tricyclic antidepressants are another example of anticholinergic drugs that may be problematic in cognitively impaired individuals [1, 21]. Instead, selective serotonin reuptake inhibitors (SSRIs) are generally the first recommended antidepressant in major NCD against both depressive symptoms and anxiety, or as an alternative to antipsychotics in the management of agitation and aggression [22]. Antidementia drugs (i.e. cholinesterase inhibitors and memantine) represent another class of psychotropics, highly relevant in major NCD. These drugs are not associated with the same risk as those previously described; on the contrary, it is desirable that treatment with cholinesterase inhibitors is routinely considered and evaluated in the management of Alzheimer’s disease (AD) [20].

The Swedish registry for cognitive/dementia disorders (SveDem) started in 2007, and the number of registrations is continuously growing with almost 90,000 people registered by the end of 2019 [23]. In brief, the registry enables an important means for improving the quality of care for people with major NCD and provides information on various factors related to cognitive function, care level and overall functional state. Baseline registrations are carried out when people are newly diagnosed, either by primary care centres or different types of specialized care units, and follow-ups are often conducted on a yearly basis.

In this study, we aim to describe psychotropic drug use and associated factors in older Swedish people diagnosed with major NCD. The objectives are to estimate psychotropic drug use through dispensation data and explore the relationships between different classes of psychotropic drugs and major NCD subtypes.

## Method

In this cross-sectional study, SveDem and the Swedish Prescribed Drug Register were linked through personal identity numbers, i.e. birth date followed by a unique four-digit number, to obtain information regarding psychotropic drugs among older people with major NCD.

### Study population

Initially, all persons ≥ 65 years registered in SveDem and with diagnosis dates from 1 May 2007 to 31 December 2017 were selected (*n* = 71,298), but only those who were alive on 31 December 2017 according to the Swedish Cause of Death Register were included in the analyses (*n* = 38,251). The study population comprised individuals living in ordinary housing and people in nursing homes or other forms of residential care.

### Outcomes and independent variables

Our estimate of psychotropic drug use was based on dispensation data from Swedish community pharmacies. These data were recorded in the Swedish Prescribed Drug Register, from which we retrospectively gathered information on filled prescriptions for psychotropics within 6 months (1 July to 31 December 2017). Drug use was defined as one or more filled prescriptions during this period. This procedure was applied on substance level and then summarized for subgroups according to the Anatomical Therapeutic Chemical (ATC) Classification System. The ATC subgroups were antipsychotics (N05A), anxiolytics (N05B), sedatives and hypnotics (N05C), antidepressants (N06A) and antidementia drugs (N06D). In the appendix (eTable 1), we conducted a sensitivity analysis with a time frame of 4 months. Information regarding the date of diagnosis, test scores and subtype of major NCD was based on first-time registrations to SveDem. The NCD subtypes in SveDem had been translated as early-onset AD, late-onset AD, vascular dementia (VaD), mixed AD and VaD, dementia with Lewy bodies (DLB), frontotemporal dementia, Parkinson’s disease dementia (PDD), unspecified dementia and other dementias. These categories corresponded to specific codes in the 10th revision of the International Statistical Classification of Diseases and Related Health Problems (ICD-10). In a population of 21,885 people where cases of mixed, unspecified and other dementias had been excluded, we explored the associations between psychotropic drug use and four simplified major NCD subtypes during the same 6-month period. These subtypes were non-mixed type AD, regardless of onset; frontotemporal dementia; non-mixed type VaD; and major NCD subtypes associated with Lewy body pathology, which could be either DLB or PDD, and for which we applied the term Lewy body dementia (LBD).

### Statistics

IBM SPSS Statistics version 25 and SAS Enterprise Guide 7.1 were used for all handling, calculations and analyses of data. We considered *p*-values < 0.05 significant for all statistical tests, and odds ratios (ORs) were calculated with 95% confidence intervals (CIs). Sex differences in proportions of individuals with filled prescriptions for psychotropics were analysed using the Pearson chi-square test. We applied multiple logistic regression to analyse the associations between ATC subgroups of psychotropics and major NCD subtypes. The adjusted regression models also included the following independent variables: age, sex, years since diagnosis and baseline Mini-Mental State Examination (MMSE) score. We assessed the linearity between independent continuous variables and the logarithms of the odds through visual inspection. In the appendix, the following analyses were performed: regression coefficients, standard errors and pseudo-R-squared measures (eTable 2); Pearson correlation matrix (eTable 3); crude ORs (eTable 4); and simple logistic regression regarding unspecified, mixed and other dementias (eTable 5).

### Ethical considerations

The study was approved by the Regional Ethical Review Board in Umeå, Sweden (registration number 2017–256-31 M). Participants in SveDem have voluntarily registered after being provided with information that the data will be used for group-level analysis within the frameworks of research and quality improvement activities. After inter-linking the SveDem-data with the Swedish Prescribed Drug Register, personal identification numbers were removed by the National Board of Health and Welfare. All individual personal data were stored in locked archives before, during and after the statistical analysis, and we only present information regarding medications, type of NCD and personal characteristics at the group level.

## Results

**Fig. 1:**
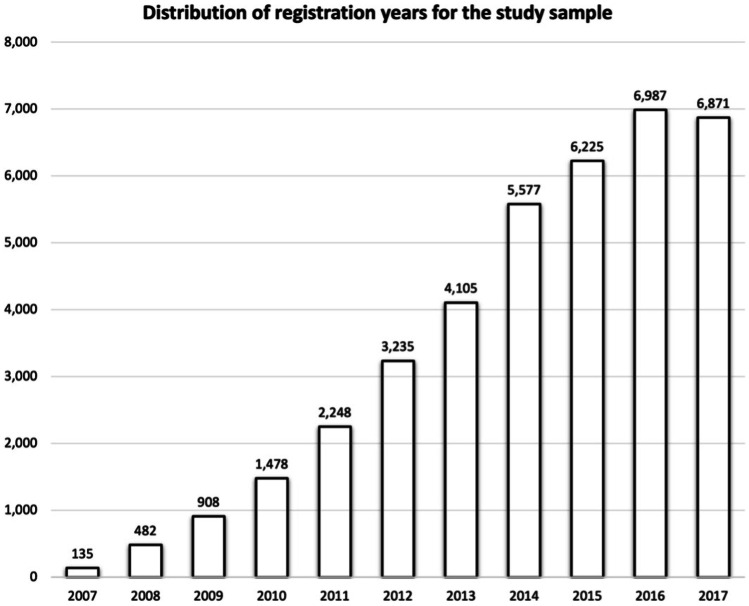
Distribution of the study population (*n* = 38,251) based on their year of being registered to the Swedish registry for cognitive/dementia disorders

The majority of the study population were registered from 2015 onwards (Fig. 1). Population characteristics and distribution of major NCD subtypes are presented in Table 1, and proportions of individuals with filled prescriptions for psychotropics are summarized in Table 2. In brief, the five most common psychotropic drugs were memantine, donepezil, mirtazapine, oxazepam and zopiclone, all with prevalence estimates in the vicinity of 20% or above. Regarding antipsychotics, risperidone had been dispensed to the highest proportion of individuals, several times more than haloperidol. Moreover, apart from haloperidol, most antipsychotics were second-generation. Changing the time frame from 6 to 4 months did not proportionally decrease the percentages of psychotropics (eTable 1), a result that was consistent for all ATC subgroups. When investigating male and female sex separately, the proportions using N05A, N05B, N05C, N06A, N06D or any psychotropic among females were 12.2%, 24.1%, 24.1%, 46.3%, 56.6% and 84.3%, respectively. The corresponding percentages for male sex were 11.7% (*p* = 0.133), 18.8% (*p* < 0.001), 21.2% (*p* < 0.001), 37.7% (*p* < 0.001), 56.9% (*p* = 0.498) and 80.7% (*p* < 0.001).

**Table 1:** Population characteristics for selected people from SveDem

Population characteristics	All selected individuals	Individuals selected for the regression analysis
Number of individuals, *n*	38,251	21,885
Female sex, % (*n*)^a^	61.4 (23,476)	61.0 (13,359)
Age (mean ± *SD*)^b^	82.5 ± 6.7	81.8 ± 6.7
Median age (IQR)	83 (9)	82 (10)
Early onset AD, % (*n*)	1.9 (729)	3.3 (729)
Late onset AD, % (*n*)	32.9 (12,595)	57.6 (12,595)
Mixed AD and VaD, % (*n*)	18.7 (7141)	n/a
VaD, % (*n*)	18.0 (6900)	31.5 (6900)
DLB, % (*n*)	1.8 (674)	3.1 (674)
PDD, % (*n*)	1.3 (488)	2.2 (488)
Frontotemporal dementia, % (*n*)	1.3 (499)	2.3 (499)
Unspecified, % (*n*)	21.6 (8272)	n/a
Other dementia type, % (*n*)	2.5 (953)	n/a
Years since diagnosis (mean)	2.8	2.8
Baseline MMSE score (mean)^c^	21.5	21.7

*AD*, Alzheimer’s disease; *DLB*, dementia with Lewy bodies; *MMSE*, Mini-Mental State Examination; *PDD*, Parkinson’s disease dementia; *SveDem*, Swedish registry for cognitive/dementia disorders; *VaD*, vascular dementia; *SD*, standard deviation; *IQR*, interquartile range.

^a^Missing data in 13 and 8 cases, respectively.

^b^Age refers to number of birthdays.

^c^Missing information in 1696 cases and 844 cases, respectively.

**Table 2:** Proportions of psychotropic drug users

Psychotropic drugs	Individuals with filled prescriptions, 1 July to 31 December 2017, % (*n*)
*Any psychotropic drug*	82.9 (31,712)
*Psychotropic drugs from* > *1 ATC-subgroup*^a^	27.9 (10,687)
*Psychotropic drugs from* > *2 ATC-subgroups*^a^	10.2 (3911)
*Psychotropic drugs from* > *3 ATC-subgroups*^a^	2.0 (781)
*Antipsychotics (N05A)*	12.0 (4594)
Risperidone	7.2 (2762)
Quetiapine	1.7 (662)
Haloperidol	1.5 (569)
Olanzapine	1.4 (530)
*Anxiolytics (N05B)*	22.0 (8430)
Oxazepam	19.9 (7626)
Hydroxyzine	1.4 (526)
Diazepam	1.1 (428)
*Sedatives or hypnotics (N05C)*	23.0 (8783)
Zopiclone	16.0 (6124)
Clomethiazole	4.0 (1525)
Zolpidem	2.6 (986)
Melatonin	2.0 (774)
Propiomazine	1.0 (400)
*Antidepressants (N06A)*	43.2 (16,511)
Mirtazapine	20.2 (7711)
Citalopram	14.0 (5366)
Sertraline	8.8 (3349)
Escitalopram	3.9 (1475)
Venlafaxine	1.8 (695)
Duloxetine	0.9 (353)
Amitriptyline	0.9 (350)
*Antidementia drugs (N06D)*	56.7 (21,686)
Memantine	26.8 (10,269)
Donepezil	25.7 (9828)
Rivastigmine	8.3 (3185)
Galantamine	6.2 (2384)

Substances with percentages < 0.5% are omitted.

^a^Subgroups N05A, N05B, N05C or N06A. Antidementia drugs are not considered.

Proportions of psychotropic drug users in terms of ATC subgroups and different subtypes of major NCD are presented in Table 3. The use of antipsychotics varied from 10.9% in VaD and late-onset AD to 25.4% in PDD. Quetiapine was more common than risperidone among people with LBD (i.e. either DLB or PDD), dispensed among 15.6% and 3.1% of the cases, respectively. The other ATC subgroups, except antidementia drugs, showed less variation between different types of major NCD. Filled prescriptions for antidepressants were found in over 40% of the study population, regardless of major NCD subtype. A more detailed investigation of the data regarding antidementia drugs in early- or late-onset AD showed that prescriptions for cholinesterase inhibitors (i.e. donepezil, rivastigmine or galantamine) had been filled in 56.9% of these cases; at least one cholinesterase inhibitor together with memantine was found in 15.1% of the population.

**Table 3:** Proportions of the study population (*n* = 38,251) who filled at least one prescription for psychotropic drugs within different major NCD subtypes

Major NCD subtype	Antipsychotics (N05A), % (*n*)	Anxiolytics (N05B), % (*n*)	Sedatives and hypnotics (N05C), % (*n*)	Antidepressants (N06A), % (*n*)	Antidementia drugs (N06D), % (*n*)
Early onset AD	14.1 (103)	21.7 (158)	19.5 (142)	44.4 (324)	76.8 (560)
Late onset AD	10.9 (1373)	21.1 (2662)	21.0 (2639)	42.3 (5329)	75.9 (9557)
Mixed AD and VaD	11.3 (810)	21.1 (1510)	25.2 (1801)	41.1 (2935)	69.1 (4932)
VaD	10.9 (755)	21.6 (1488)	25.4 (1753)	44.4 (3061)	20.5 (1417)
DLB	18.0 (121)	21.2 (143)	26.3 (177)	48.5 (327)	82.3 (555)
PDD	25.4 (124)	20.5 (100)	23.8 (116)	51.4 (251)	70.9 (346)
Frontotemporal dementia	17.0 (85)	19.0 (95)	22.6 (113)	46.5 (232)	19.2 (96)
Unspecified dementia	13.2 (1096)	24.9 (2063)	22.1 (1830)	43.8 (3627)	46.5 (3843)
Other dementia type	13.3 (127)	22.1 (211)	22.2 (212)	44.6 (425)	39.9 (380)

*AD*, Alzheimer’s disease; *DLB*, dementia with Lewy bodies; *PDD*, Parkinson’s disease dementia; *VaD*, vascular dementia.

### Multiple logistic regression analyses

**Fig. 2:**
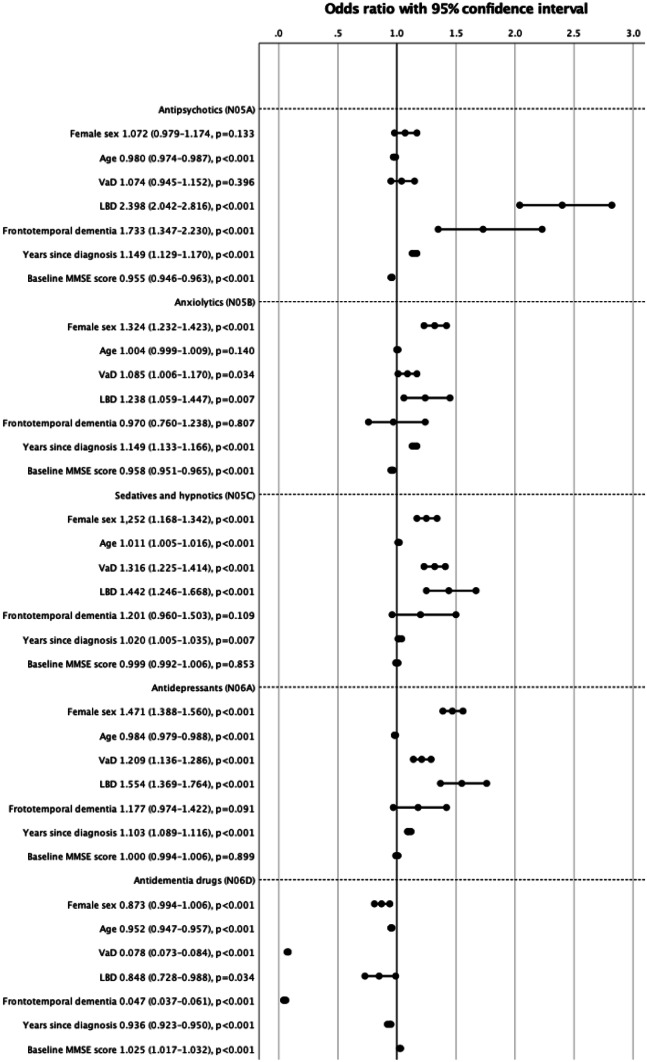
Forest plot of adjusted odds ratios by psychotropic drug class. Alzheimer’s disease was used as the reference category. Due to missing data regarding sex (*n* = 8) and MMSE score (*n* = 844), the analysis included 21,033 people. The odds ratios for age and MMSE are per year and unit of test score, respectively. LBD, Lewy body dementia; MMSE, Mini-Mental State Examination; PDD, Parkinson’s disease dementia; VaD, vascular dementia

The population selected for the logistic regression analysis of drug use in different subgroups of NCD numbered a total of 21,885 people, comprising 13,324 individuals with AD, 6900 with VaD, 1161 with LBD and 499 with frontotemporal dementia. The results from the adjusted regression models are presented in Fig. 2. In brief, filled prescriptions for psychotropic drugs were significantly more common among women for all ATC subgroups other than antipsychotics and antidementia drugs. The latter was instead negatively associated with female sex. The direction of the relationship between psychotropic drug use and age varied across the drug classes, and except for antidementia drugs, longer time since diagnosis was consistently associated with higher use of psychotropics. Among the different major NCD subtypes, LBD indicated the strongest associations with drug use compared to AD, and this observation was consistent for all ATC subgroups.

## Discussion

### Findings

When treating psychotropics as a homogenous group, our observed percentage of approximately 83% regarding the use of any psychotropic drug (Table 2) were between reported prevalence estimates of 88% [24] and 66% [25] among nursing homes residents with major NCD. Moreover, three or more drugs from different ATC groups had occurred in 10.2% of the study population (Table 2), but we cannot tell whether these results indicate actual polypharmacy or simply that one drug had replaced another. Regarding specific ATC subgroups other than antidementia drugs, most of our results seem lower than those found by Gustafsson et al. regarding people with major NCD, for example, 40% antipsychotic users in Swedish specialized residential care during 2005–2006 [9] and 19% antipsychotic users in different types of geriatric units in 2013 [26]. We found that 12.0% of the individuals had been dispensed antipsychotics (Table 2), and this variation may be due to heterogeneous study populations since institutionalized people are on average expected to be in a later and more severe stage of NCD, often with prominent NPS as a major reason for nursing home placement [27, 28]. The differences could also reflect various efforts made in Sweden to improve drug therapy, such as the quality indicators issued by the National Board of Health and Welfare that were first released in 2004 [20]. Our proportion of antipsychotic users is similar to prevalence estimates from both France [29] and the UK [30], approximately 10% and 8–10%, respectively, during similar time frames; however, those observational studies applied a more restrictive definition of drug use. On the other hand, the pooled prevalence of any antipsychotic reported by Kirkham et al. [31] was as high as 27.5% (95% CI 25.7–29.3%, *p* < 0.001). Even though our findings may not be conspicuous, there are still apparent differences between subtypes of major NCD, where LBD appears to be highly associated with the use of antipsychotics. In the present study, we found proportions of antipsychotic users to be 18.0% in DLB and 25.4% in PDD (Table 3). Similar proportions of antipsychotic drug users have been documented earlier in Sweden, for example, > 20% regarding residents with LBD features in Swedish nursing homes [32] and 16% among people with DLB in an earlier investigation of SveDem by Johnell et al. [33]. Our observations are probably due to the relatively high frequency of hallucinations and other psychotic symptoms in LBD compared to AD [3, 4, 34]. Nonetheless, the results call for concern since these individuals are extremely sensitive to antipsychotic drugs, with an increased risk of adverse drug reactions, such as malignant neuroleptic syndrome or further impairment of motor functions [35–39]. However, in our current study, it is uncertain whether antipsychotics have been utilized excessively, i.e. situations other than either schizophrenia or psychotic or severely aggressive behaviour; furthermore, there were no data regarding dosages and evaluation of treatments. On the positive side, most antipsychotics used in LBD were quetiapine, which appears to be the least unfavourable option [40]. Despite the large study population, we found no difference in antipsychotic drug use (*p* = 0.133), contrary to earlier findings by Lövheim et al. where the male and female proportions were 35.5% and 28.9% (p < 0.001), respectively [41]. Except for antidementia drugs, our data were in overall agreement with earlier observations that psychotropic drug use is more common among females [42, 43]. Previous studies have found that men and women with AD are different in terms of severity and types of NPS [44–46], but that is probably not the whole explanation. It is worrisome if systematic discrimination occurs with a lower threshold for utilizing psychotropics among women or if men are undertreated.

Zopiclone and oxazepam were frequently dispensed in the population, and these drugs are generally recommended in favour of sedating antihistamines and long-acting BZDs among older people [20, 47]. In comparison, 1.1% of the persons had redeemed prescriptions for propiomazine, a figure which is approximately half that of an earlier prevalence estimate [48]. Nevertheless, even for short–intermediate-acting BZDs and zopiclone, the associations with falls and hospitalizations may still be valid [49–53].

The proportion of antidepressant users was almost 50% (Table 2) and might indicate a transition from antipsychotics towards antidepressants in the treatment of NPS, a trend earlier observed in the UK [54]. However, depression in major NCD is highly complex and could, for example, either be a prodrome of major NCD or a reaction to the declining functions and capabilities [55], and the Swedish Prescribed Drug Register provides no information as to whether antidepressants were prescribed to treat major depression, depressive symptoms or other NPS. Of note, mirtazapine appears to be the most utilized antidepressant among older Swedish people with major NCD, whereas national treatment guidelines recommended SSRIs as the first-line pharmacological option for depression, even in cases of co-existing cognitive disorder [47]. Plausible explanations could be the increased risk of hyponatraemia associated with citalopram and other SSRI [56], potential drug–drug interactions or simply that the physicians try to utilize the potential side effects of mirtazapine for purposes of weight gain or managing sleep disorders, which are common features of the NPS spectrum [3, 4]. Still, daytime sedation is common, and the association between mirtazapine and falls among older people is, to our knowledge, an area of limited research.

The high amounts of antidementia drugs in AD might be interpreted as a vague indicator that the need to improve cognitive symptoms and self-sustainability is acknowledged; however, the results do not confirm whether everyone that may benefit from cholinesterase inhibitors is considered for such treatments. Nor is it certain to what extent these treatments are properly evaluated over time, an aspect of particular relevance beyond the point of admission to residential care or in other severe stages of major NCD where the chance of impeding cognitive decline may be less likely. On the other hand, the numbers in Fig. 1 show that most of the study population had been diagnosed within 3 years before the end of 2017, and this group could presumably comprise many cases of relatively mild or moderate AD, which are the original indications for most antidementia drugs. Cholinesterase inhibitors may, in addition to improving ratings of cognitive functions, global assessments and activities of daily living, also be beneficial for alleviating many of the characterizing and burdensome NPS in LBD [57–59], which may explain the relatively minor difference compared to AD.

### Strengths

The number of reporting primary care units had surpassed 900 (approximately 80% of all primary care centres) by the end of 2017 and had provided approximately 45% of all new registrations during that period [60]. The studied individuals should therefore be representative of the two main settings where cognitive examinations in Sweden are conducted, i.e. primary and specialized care units. Moreover, the cases of AD were similar to previous worldwide prevalence estimates [61]. The size of the data set is another important factor to consider, even though the exact coverage and representativity remain uncertain. Furthermore, dispensation data can, compared with prescription records, provide more accurate estimations regarding ongoing treatments and actual drug exposure. In Sweden, prescriptions for subsidized medicines are generally not issued for more than 3 months at a time, and dispensation data for individuals with at least 50% of days covered with drug supply for long-term treatments were therefore most likely included in our statistical presentation and analysis. Decreasing the period from 6 to 4 months, a more conservative approach to define drug users, had little impact on prescription fills (Appendix, eTable 1).

### Limitations and risk of bias

To compare well-defined categories in the regression analysis, we removed all individuals diagnosed with mixed, uncertain, or other dementia from the regression analysis, and the uncertainties regarding the large proportions of such diagnoses should be mentioned in terms of potential bias risk. It is reasonable to suspect that the unspecified cases represented a considerable number of persons with LBD, especially compared with other prevalence estimates, which suggest a figure around 5–10% [62–64] compared to our observed proportion of 3.1% (Table 1). Although less than 5% of all SveDem registrations during 2007–2017 had been changed during follow-up [60], uncommon types of major NCD, e.g. LBD and frontotemporal dementia, may in many cases still have been labelled as unspecified dementia during the initial primary care examination and instead been referred to specialized care units for a re-evaluation of the diagnosis. Missing data regarding the MMSE result and the deletion of those individuals in the multiple regression models is another potential source of bias that should be considered when interpreting the adjusted ORs, especially since those cognitive evaluations might have been more of a standard procedure in certain reporting units, i.e. specialized versus primary care.

In nursing homes and similar institutions, insufficient staff and caregiver distress may be barriers against the implementation of more rational non-pharmacological alternatives to antipsychotics [25, 65–67], but we had no information about NPS and nursing home placement for the time period of interest. Among other potential confounders, socioeconomic disadvantage deserves to be mentioned [68], and although there are many aspects of socioeconomic status, this factor is perhaps less important in Sweden where most prescription medications are covered by a subsidiary system with a maximum 12-month cost of approximately 2350 SEK. Another limitation is that our definition of drug use did not capture dosage regimen, total drug supply, indication for therapy and treatment duration, and these factors should ideally be given more focus to better assess the appropriateness of medication use.

## Conclusions

Psychotropic drugs were frequently dispensed among older Swedish people with major NCD. The percentages of antipsychotics and drugs with sedative properties are especially relevant from a risk perspective. The association between antipsychotic drug use and people with LBD warrants particular concern since these individuals are highly sensitive to adverse drug reactions from such medications. Moreover, antidepressants and antidementia drugs had been dispensed in about half of the study population. Despite being potential indicators of deliberate and rational medication use, these long-term treatments still require continuous evaluation regarding effect versus safety. More restrictive prescribing of psychotropics may further reduce drug-related problems among individuals with major NCD; however, longitudinal and comparative approaches are needed to better assess the appropriateness of psychotropic drug use in this vulnerable group of people.

## Supplementary Information

Below is the link to the electronic supplementary material.

Supplementary file1 (PDF 317 KB)

## Data Availability

The datasets generated during and/or analysed in this study are available from the corresponding author upon reasonable request.
