# Supplementary material for: Psychotropic drug use among older people with major neurocognitive disorder: a cross-sectional study based on Swedish national registries
Source: Eur J Clin Pharmacol. 2021 Nov 4;78(3):477–87. doi: 10.1007/s00228-021-03241-7 (PMC8818624; doi:10.1007/s00228-021-03241-7)
Supplement: Supplementary file 1 — Supplementary file1 (PDF 317 KB) [file 228_2021_3241_MOESM1_ESM.pdf]

# Appendix – Supplementary statistics

Accompanying the manuscript:

Psychotropic drug use among older people with major neurocognitive disorder: a cross-sectional study based on Swedish national registries

European Journal of Clinical Pharmacology

Jonas Kindstedt, Maria Sjölander, Hugo Lövheim and Maria Gustafsson

Affiliation and e-mail of the corresponding author: Department of Integrative Medical Biology (IMB), Umeå University, Umeå, Sweden
jonas.kindstedt@umu.se

Content

eTable 1. Proportions of individuals with filled prescriptions for psychotropic drugs using a 4-month time frame.

eTable 2. Adjusted odds ratios and supplementary statistical output from the multiple logistic regression models regarding population characteristics and psychotropic drugs.

eTable 3. Bivariate Pearson correlation matrix for continuous covariates in the regression models.

eTable 4. Crude ORs from the simple logistic regression analyses regarding population characteristics and psychotropic drugs.

eTable 5. Supplementary logistic regression analyses regarding psychotropic drugs and mixed, unspecified and other dementia.

**eTable 1.** Proportions of individuals with filled prescriptions for psychotropic drugs using a 4-month time frame.

| Psychotropic drugs | Individuals with filled prescriptions 1 September–31 December 2017 % (n)[a] |
|---|---|
| *All psychotropic drugs* | 81.3 (31,096) |
| *Antipsychotics (N05A)* | 11.5 (4,402) |
| Risperidone | 6.9 (2,637) |
| Quetiapine | 1.7 (632) |
| Haloperidol | 1.3 (508) |
| Olanzapine | 1.3 (506) |
| *Anxiolytics (N05B)* | 11.5 (4,402) |
| Oxazepam | 18.1 (6,919) |
| Hydroxyzine | 1.2 (444) |
| Diazepam | 0.9 (349) |
| *Sedatives or hypnotics (N05C)* | 21.3 (8,129) |
| Zopiclone | 14.8 (5,657) |
| Clomethiazole | 3.5 (1,335) |
| Zolpidem | 2.3 (877) |
| Melatonin | 1.9 (708) |
| Propiomazine | 0.9 (342) |
| *Antidepressants (N06A)* | 42.0 (16,066) |
| Mirtazapine | 19.6 (7,497) |
| Citalopram | 13.5 (5,182) |
| Sertraline | 8.5 (3,235) |
| Escitalopram | 3.7 (1,409) |
| Venlafaxine | 1.7 (668) |
| Duloxetine | 0.9 (334) |
| Amitriptyline | 0.9 (326) |
| *Antidementia drugs (N06D)* | 55.0 (21,037) |
| Memantine | 26.0 (9,960) |
| Donepezil | 24.6 (9,406) |
| Rivastigmine | 7.8 (2,991) |
| Galantamine | 6.0 (2,312) |

[a] Results less than 0.5% are omitted from the presentation.

**eTable 2.** Adjusted odds ratios and supplementary statistical output from the multiple logistic regression models regarding population characteristics and psychotropic drugs.

| | OR | CI (95%) | P-value | B | S.E |
|---|---|---|---|---|---|
| *Antipsychotics – N05A* | | | | | |
| Female[a] | 1.072 | 0.979–1.174 | 0.133 | 0.070 | 0.046 |
| Age | 0.980 | 0.974–0.987 | < 0.001 | -0.020 | 0.003 |
| VaD[b] | 1.044 | 0.945–1.152 | 0.396 | 0.043 | 0.051 |
| LBD[b] | 2.398 | 2.042–2.816 | < 0.001 | 0.875 | 0.082 |
| Frontotemporal dementia[b] | 1.733 | 1.347–2.230 | < 0.001 | 0.550 | 0.129 |
| Years since diagnosis | 1.149 | 1.129–1.170 | < 0.001 | 0.139 | 0.009 |
| Baseline MMSE score[c] | 0.955 | 0.946–0.963 | < 0.001 | -0.046 | 0.005 |
| *Anxiolytics – N05B* | | | | | |
| Female sex[a] | 1.324 | 1.232–1.423 | < 0.001 | 0.281 | 0.037 |
| Age | 1.004 | 0.999–1.009 | 0.140 | 0.004 | 0.003 |
| VaD[b] | 1.085 | 1.006–1.170 | 0.034 | 0.081 | 0.038 |
| LBD[b] | 1.238 | 1.059–1.447 | 0.007 | 0.213 | 0.080 |
| Frontotemporal dementia[b] | 0.970 | 0.760–1.238 | 0.807 | -0.030 | 0.124 |
| Years since diagnosis | 1.149 | 1.133–1.166 | < 0.001 | 0.139 | 0.007 |
| Baseline MMSE score[c] | 0.958 | 0.951–0.965 | < 0.001 | -0.043 | 0.004 |
| *Sedatives and hypnotics – N05C* | | | | | |
| Female sex[a] | 1.252 | 1.168–1.342 | < 0.001 | 0.225 | 0.035 |
| Age | 1.011 | 1.005–1.016 | < 0.001 | 0.010 | 0.003 |
| VaD[b] | 1.316 | 1.225–1.414 | < 0.001 | 0.275 | 0.037 |
| LBD[b] | 1.442 | 1.246–1.668 | < 0.001 | 0.366 | 0.074 |
| Frontotemporal dementia[b] | 1.201 | 0.960–1.503 | 0.109 | 0.183 | 0.114 |
| Years since diagnosis | 1.020 | 1.005–1.035 | 0.007 | 0.020 | 0.007 |
| Baseline MMSE score[c] | 0.999 | 0.992–1.006 | 0.853 | -0.001 | 0.004 |
| *Antidepressants (N06A)* | | | | | |
| Female sex[a] | 1.471 | 1.388–1.560 | < 0.001 | 0.386 | 0.030 |
| Age | 0.984 | 0.979–0.988 | < 0.001 | 0.016 | 0.002 |
| VaD[b] | 1.209 | 1.136–1.286 | < 0.001 | 0.190 | 0.032 |
| LBD[b] | 1.554 | 1.369–1.764 | < 0.001 | 0.441 | 0.065 |
| Frontotemporal dementia[b] | 1.177 | 0.974–1.422 | 0.091 | 0.163 | 0.096 |
| Years since diagnosis | 1.103 | 1.089–1.116 | < 0.001 | 0.098 | 0.006 |
| Baseline MMSE score[c] | 1.000 | 0.994–1.006 | 0.899 | 0.000 | 0.003 |
| *Antidementia drugs (N06D)* | | | | | |
| Female sex[a] | 0.873 | 0.994–1.006 | < 0.001 | -0.136 | 0.035 |

| | | | | | |
|---|---|---|---|---|---|
| Age | 0.952 | 0.947–0.957 | < 0.001 | -0.049 | 0.003 |
| VaD[b] | 0.078 | 0.073–0.084 | < 0.001 | -2.548 | 0.038 |
| LBD[b] | 0.848 | 0.728–0.988 | 0.034 | -0.165 | 0.078 |
| Frontotemporal dementia[b] | 0.047 | 0.037–0.061 | < 0.001 | -3.049 | 0.125 |
| Years since diagnosis | 0.936 | 0.923–0.950 | < 0.001 | -0.066 | 0.007 |
| Baseline MMSE score[c] | 1.025 | 1.017–1.032 | < 0.001 | 0.024 | 0.004 |

Cox and Snell R-squared values for the regression model were 0.019 (N05A), 0.026 (N05B), 0.006 (N05C), 0.022 (N06A) and 0.276 (N06D).

B, regression coefficient (Beta); CI, confidence interval; LBD, Lewy body dementia; MMSE, Mini Mental State Exam; OR, odds ratio; S.E, standard error, VaD, vascular dementia

[a] Data were missing for 8 individuals
[b] Alzheimer's disease was used as the reference category
[c] Data were missing for 844 individuals

**eTable 3.** Bivariate Pearson correlation matrix for continuous covariates in the regression models.

| | | Age | Years since diagnosis | Baseline MMSE score |
|---|---|---|---|---|
| Age | Coefficient | 1 | 0.174 | -0.080 |
| | P-value | n/a | <0.011 | <0.001 |
| Years since diagnosis | Coefficient | 0.174 | 1 | 0.149 |
| | P-value | <0.001 | n/a | <0.001 |
| Baseline MMSE score | Coefficient | -0.080 | 0.149 | 1 |
| | P-value | <0.001 | <0.001 | n/a |

Individuals with missing data regarding MMSE results (n=844) were excluded listwise.

MMSE, Mini Mental State Examination

**eTable 4.** Crude ORs from the simple logistic regression analyses regarding population characteristics and psychotropic drugs.

| | Simple logistic regression | | |
|---|---|---|---|
| | **OR** | **CI (95%)** | **P-value** |
| *Antipsychotics (N05A)* | | | |
| Female | 1.048 | 0.963–1.141 | 0.278 |
| Age | 0.987 | 0.981–0.993 | < 0.001 |
| VaD[a] | 0.986 | 0.899–1.082 | 0.770 |
| LBD[a] | 2.145 | 1.844–2.494 | < 0.001 |
| Frontotemporal dementia[a] | 1.648 | 1.297–2.094 | < 0.001 |
| Years since diagnosis | 1.111 | 1.093–1.130 | < 0.001 |
| Baseline MMSE score | 0.968 | 0.959–0.976 | < 0.001 |
| *Anxiolytics (N05B)* | | | |
| Female sex | 1.389 | 1298–1.488 | < 0.001 |
| Age | 1.019 | 1.014–1.024 | < 0.001 |
| VaD[a] | 1.024 | 0.954–1.099 | 0.510 |
| LBD[a] | 0.985 | 0.850–1.141 | 0.840 |
| Frontotemporal dementia[a] | 0.876 | 0.698–1.099 | 0.253 |
| Years since diagnosis | 1.135 | 1.120–1.151 | < 0.001 |
| Baseline MMSE score | 0.967 | 0.960–0.974 | < 0.001 |
| *Sedatives and hypnotics (N05C)* | | | |
| Female sex | 1.209 | 1.132–1.291 | < 0.001 |
| Age | 1.014 | 1.009–1.019 | < 0.001 |
| VaD[a] | 1.291 | 1.206–1.383 | < 0.001 |
| LBD[a] | 1.278 | 1.113–1.469 | 0.001 |
| Frontotemporal dementia[a] | 1.110 | 0.896–1.374 | 0.339 |
| Years since diagnosis | 1.024 | 1.010–1.037 | 0.001 |
| Baseline MMSE score | 0.998 | 0.991–1.005 | 0.503 |
| *Antidepressants – N06A* | | | |
| Female sex | 1.413 | 1.337–1.493 | < 0.001 |
| Age | 0.994 | 0.990–0.998 | 0.002 |
| VaD[a] | 1.082 | 1.020–1.147 | 0.008 |
| LBD[a] | 1.343 | 1.191–1.514 | < 0.001 |
| Frontotemporal dementia[a] | 1.179 | 0.986–1.411 | 0.072 |
| Years since diagnosis | 1.092 | 1.080–1.105 | < 0.001 |
| Baseline MMSE score | 1.007 | 1.001–1.013 | 0.022 |
| *Antidementia drugs – N06D* | | | |
| Female sex | 1.015 | 0.961–1.072 | 0.593 |

| | | | |
|---|---|---|---|
| Age | 0.95 | 0.945–0.949 | < 0.001 |
| VaD[a] | 0.082 | 0.076–0.088 | < 0.001 |
| LBD[a] | 1.094 | 0.948–1.263 | 0.218 |
| Frontotemporal dementia[a] | 0.08 | 0.076–0.095 | < 0.001 |
| Years since diagnosis | 0.969 | 0.958–0.980 | < 0.001 |
| Baseline MMSE score | 1.025 | 1.019–1.031 | < 0.001 |

[a] Alzheimer's disease was used as the reference category

**eTable 5.** Supplementary logistic regression analyses regarding psychotropic drugs and mixed, unspecified and other dementia.

| | **Simple logistic regression** | | |
|---|---|---|---|
| | **OR** | **CI (95%)** | **P-value** |
| *Antipsychotics (N05A)* | | | |
| Mixed AD and VaD | 1.027 | 0.938–1.125 | 0.566 |
| Unspecified dementia | 1.226 | 1.128–1.333 | < 0.001 |
| Other dementia | 1.234 | 1.016–1.499 | 0.034 |
| *Anxiolytics (N05B)* | | | |
| Mixed AD and VaD | 0.999 | 0.931–1.072 | 0.974 |
| Unspecified dementia | 1.238 | 1.160–1.321 | < 0.001 |
| Other dementia | 1.059 | 0.904–1.241 | 0.477 |
| *Sedatives and hypnotics (N05C)* | | | |
| Mixed AD and VaD | 1.279 | 1.195–1.368 | < 0.001 |
| Unspecified dementia | 1.077 | 1.008–1.151 | 0.029 |
| Other dementia | 1.085 | 0.926–1.271 | 0.314 |
| *Antidepressants (N06A)* | | | |
| Mixed AD and VaD | 0.947 | 0.893–1.004 | 0.067 |
| Unspecified dementia | 1.060 | 1.003–1.120 | 0.041 |
| Other dementia | 1.092 | 0.957–1.247 | 0.191 |
| *Antidementia drugs (N06D)* | | | |
| Mixed AD and VaD | 0.708 | 0.664–0.755 | < 0.001 |
| Unspecified dementia | 0.275 | 0.259–0.292 | < 0.001 |
| Other dementia | 0.210 | 0.184–0.241 | < 0.001 |

Crude ORs for each subtype of major neurocognitive disorder and psychotropic drug class are displayed. The same 29,690 individuals were included in all regression models. In all models, AD was used as the reference category

AD, Alzheimer's disease; CI, confidence interval; OR, odds ratio; VaD, vascular dementia
